# First detection of African swine fever (ASF) virus genotype X and serogroup 7 in symptomatic pigs in the Democratic Republic of Congo

**DOI:** 10.1186/s12985-020-01398-8

**Published:** 2020-09-03

**Authors:** Patrick N. Bisimwa, Juliette R. Ongus, Christian K. Tiambo, Eunice M. Machuka, Espoir B. Bisimwa, Lucilla Steinaa, Roger Pelle

**Affiliations:** 1 Department of Molecular Biology and Biotechnology, Pan African University, Institute of Basic Sciences, Technology and Innovation, Nairobi, Kenya; 2 Department of Animal Science and Production, Université Evangélique en Afrique, Bukavu, Democratic Republic of the Congo; 3 Department of Medical Laboratory Sciences, Jomo Kenyatta University of Agriculture and Technology, Juja, Kenya; 4 Biosciences eastern and central Africa-International Livestock Research Institute (BecA-ILRI) Hub, Naivasha Road, P.O. Box 30709, Nairobi, 00100 Kenya; 5 Centre for Tropical Livestock Genetics and Health (CTLGH), International Livestock Research Institute, Nairobi, Kenya; 6 International Livestock Research Institute, Animal and Human Health, Nairobi, Kenya

**Keywords:** African swine fever virus, Domestic pigs, Phylogeny, Genotype-serogroup, South Kivu province, DR Congo

## Abstract

**Background:**

African swine fever (ASF) is a highly contagious and severe hemorrhagic viral disease of domestic pigs. The analysis of variable regions of African swine fever virus (ASFV) genome led to more genotypic and serotypic information about circulating strains. The present study aimed at investigating the genetic diversity of ASFV strains in symptomatic pigs in South Kivu province of the Democratic Republic of Congo (DRC).

**Materials and methods:**

Blood samples collected from 391 ASF symptomatic domestic pigs in 6 of 8 districts in South Kivu were screened for the presence of ASFV, using a *VP73* gene-specific polymerase chain reaction (PCR) with the universal primer set PPA1-PPA2. To genotype the strains, we sequenced and compared the nucleotide sequences of PPA-positive samples at three loci: the C-terminus of *B646L* gene encoding the p72 protein, the *E183L* gene encoding the p54 protein, and the central hypervariable region (CVR) of the *B602L* gene encoding the J9L protein. In addition, to serotype and discriminate between closely related strains, the *EP402L* (CD2v) gene and the intergenic region between the *I73R* and *I329L* genes were analyzed.

**Results:**

ASFV was confirmed in 26 of 391 pigs tested. However, only 19 and 15 PPA-positive samples, respectively, were successfully sequenced and phylogenetically analyzed for p72 (*B646L*) and p54 (*E183L*). All the ASFV studied were of genotype X. The CVR tetrameric repeat clustered the ASFV strains in two subgroups: the Uvira subgroup (10 TRS repeats, AAAABNAABA) and another subgroup from all other strains (8 TRS repeats, AABNAABA). The phylogenetic analysis of the *EP402L* gene clustered all the strains into CD2v serogroup 7. Analyzing the intergenic region between *I73R* and *I329L* genes revealed that the strains were identical but contained a deletion of a 33-nucleotide internal repeat sequence compared to ASFV strain Kenya 1950.

**Conclusion:**

ASFV genotype X and serogroup 7 was identified in the ASF disease outbreaks in South Kivu province of DRC in 2018–2019. This represents the first report of ASFV genotype X in DRC. CVR tetrameric repeat sequences clustered the ASFV strains studied in two subgroups. Our finding emphasizes the need for improved coordination of the control of ASF.

## Background

Pigs are increasingly contributing to improved nutrition and household incomes in regions of Africa where pork consumption and pig keeping are culturally acceptable [1]. Despite the importance of pig farming, this sector is facing several constraints, with infectious disease burden being the major problem [2]. African swine fever virus (ASFV) causes an acute, highly contagious, and fatal disease in domestic pigs, with clinical signs such as fever and haemorrhagic lesions [3]. There are currently no vaccines available to combat African swine fever (ASF). The first recorded ASF outbreaks were reported in pigs belonging to European settlers in Kenya in 1914 [4]. The disease continues to spread throughout Eastern Europe since 2007 [5] and was reported in Belgium and China in 2018 [6–8]. ASFV is a large, enveloped, double-stranded DNA arbovirus belonging to the genus *Asfivirus,* and the only member of the family *Asfarviridae* [2, 9, 10]. Warthogs, bush pigs and the soft tick of the genus *Ornithodoros* are the major reservoirs of ASFV [3]. The contagious nature and the ability to spread rapidly in pig populations over long distances makes it the most feared disease of domestic pigs [11, 12]. The genome size varies from 170 to 193 kbp and encodes between 150 and 167 open reading frames, depending on the virus strains [13]. To date, 24 ASFV genotypes have been reported worldwide based on the *B646L* gene, which encodes the capsid protein p72, and all of them are known to circulate in Africa [14–16]. Using the serotype-identification approach [17], an additional 8 ASFV serotypes have been reported based on the *EP402R* gene encoding the CD2V protein [17–19]. Distinct antigenic types of ASFV were identified based on haemadsorption inhibition (HAI) serological typing, where ASF protective immunity was shown to be serotype-specific, and viruses belonging to identical serogroups cross-protected against each other [20]. This has significant importance for vaccine development. The CD2V protein, encoded by the ASFV EP402R gene, is a transmembrane glycoprotein located in the viroplasm (around viral factories) and in the plasma membrane of infected cells. It is among the most variable genes in the ASFV genome [21, 22]. Haemadsorption involves adhesion of pig erythrocytes to the surface of ASFV infected cells, a key requirement is expression of CD2v in ASFV-infected cells [20].

Control of the disease is relying on surveillance, restriction of pigs and pork products movement, and rapid diagnosis and culling of ASFV infected animals. The implementation of these measures is particularly difficult for African pig farmers of which many can be characterized as smallholders, due to limited capacity and appropriate policy. In 2011, ASF outbreaks were reported in more than 25 African countries with the highest number of outbreaks (84) registered in the Democratic Republic of Congo (DRC) causing a loss of 105,614 pigs [23, 24]. Previous studies have reported circulation of genotype I, IX and XIV in DRC, encouraging the need for continued characterization of ASFV strains responsible for outbreaks to better understand the spread of this economical important disease in DRC. Several variable regions of the ASFV genome have been extensively used as targets for molecular epidemiology studies of ASFV strains [25–27]. However, previous studies achieved high resolution for discrimination between different virus strains when combining *P72, P54* and *B602L* (Central Variable Region or CVR) proteins [28, 29]. Moreover, the *EP402R* gene encoding the CD2v protein and the intergenic region between the *I73R* and *I329L* have also been used to characterize ASFV from various locations and to track virus spread [25, 26]. The first report of the presence of ASF in DRC was in 1939 [30]. South Kivu province is an area in the eastern DRC where suspected cases of ASF appear regular. Reports from the Provincial Ministry of Agriculture Livestock and Fishery (PMALF) and the local veterinary body indicated the death of 1600 pigs out of 1608 that presented clinical signs of ASF in 2015 (Report of the PMALF, unpublished data). More recently, we have used a combination of *P72* and *P54* proteins to characterize ASFV genotype IX in apparently healthy pigs in South Kivu province sampled in 2016 [31]. It was the first study of ASFV in the South Kivu province. However, despite report of frequent incidences of ASF in the South Kivu province by the PMALF, information about ASFV strains in circulation in suspected infected animals is lacking. Therefore, the present study was set up to identify and characterize ASFV strains in infected domestic pigs with symptoms of ASF from different smallholder farms in the South Kivu province in order to increase epidemiological knowledge of ASFV, and to generate information for improvement of control strategies.

## Materials and methods

### Ethics statement

Ethical approval for the study reported here and the permission for the collection of samples was provided by the Interdisciplinary Centre for Ethical Research (CIRE) established by the Evangelical University in Africa, Bukavu, DR Congo, with reference (UEA/SGAC/KM 132/2016). A consent form which described the aim of the study was signed by farmers willing to participate in the study after translation into local languages.

### Study area

**Fig. 1:**
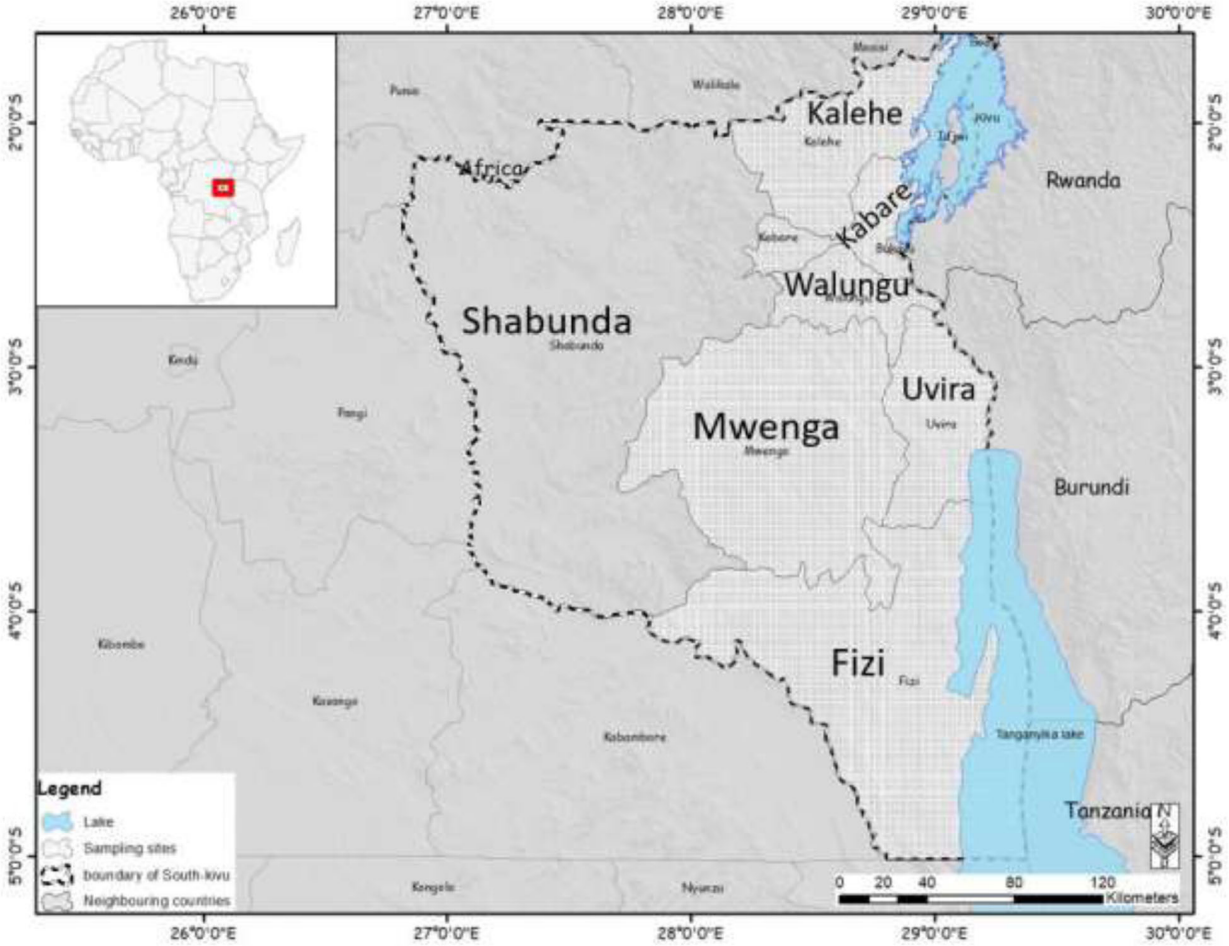
Map of South Kivu in the Democratic Republic of Congo showing the sampling areas

The study was carried out in South Kivu province of the Democratic Republic of Congo (DRC), situated in the eastern part of the country. It is a large region with an area of 66,814 km^2^, located between longitudes 26° 10′ 30″ and 29° 58′ east, latitudes 00′ 58″ and 4° 51′ 21″ South. Comparatively, the South Kivu province is over two times the size of Burundi (27,834 km^2^) and Rwanda (26,338 km^2^) put together. The province experiences two main seasons: a 9-month long rainy period, from September to May, and a 3-month dry period (June to August). The annual average rainfall is 1300 mm. Six out of eight territories were selected for purposes of this study including; Fizi, Kabare, Kalehe, Mwenga, Uvira and Walungu (Fig. 1). A key factor in selecting the sample sites was the inclusion of the main pig-producing, marketing, and consuming areas, with a particular focus on locations where suspected ASF outbreaks had been recently reported by the Provincial Ministry of Agriculture Livestock and Fishery.

### Study design and sample collection

A cross-sectional study was conducted where the target population was households that keep pigs, and where suspected ASF cases were notified based on the reports from the local veterinary and Provincial Ministry of Agriculture Livestock and Fishery during December 2018 to January 2019. In general, the number of pigs per farm varied between 1 and 5 and they shared housing. Thus, farms with pigs presenting symptoms such as high fever, reddening of the skin, particularly ears and snout, coughing and difficulties in breathing, hemorrhagic diarrhea and vomiting, inability to walk, loss of appetites, general weakness, were considered for sampling and all the pigs were sampled. DRC has a pig population of approximately 1 million. The recommended sample size for a population of that size, using a confidence level of 95% and a margin of error of 5% would be 384 [32]. Based on this, a total of 391 blood samples from suspected ASFV infected pigs were collected in EDTA (anticoagulant) tubes and were used for PCR analysis. All blood samples were collected from the jugular vein of pigs of over 3 months of age. After collection, all collected blood samples were transported to the Université Evangélique en Afrique (UEA) and stored at − 20 °C before being shipped on ice packs to the Pan African University Institute of Science Technology and Innovation (PAUISTI) in Nairobi then to BecA-ILRI Hub, for subsequent analysis.

### DNA extraction and ASFV detection

Total DNA was extracted directly from 200 μl of whole blood using the DNeasy Blood and Tissue Kit (Qiagen, USA) following the manufacturer’s recommendations. To detect the presence of ASFV DNA, polymerase chain reaction (PCR) amplification assay was carried out using the ASFV diagnosis primers PPA1/PPA2 (Peste Porcine Africaine) that target the virus *VP73* (p72) coding region to generate an amplicon of 257 bp [33]. PCR products were confirmed using a 2% agarose gel electrophoresis. All PPA-positive samples were characterized in subsequent analyses.

### PCR genotyping and sequence analysis

Five separate PCR experiments were set up to amplify PPA-positive samples: (i) for p72 genotype classification, the C-terminal region of the p72 protein gene was amplified using the primers p72-U/D [15]; (ii) for p54 genotyping, the gene *E183L* encoding the p54 protein was amplified using the primers PPA722/PPA89 [11]; (iii) the *B602L* gene characterized by the Central Variable Region (CVR) was amplified using the primer pairs CVR-FL1 and CVR–FL2 as previously reported [24]; (iv) for determining the origin, and to distinguish between closely related ASFV strains circulating in the South Kivu province, a 356 bp fragment, specific for identification of Tandem Repeat Sequences (TRS), located between the *I73R* and *I329L* genes was amplified using the primer pairs ECO1A and ECO1B [34]; (v) to determine the serogroups of the strains, the partial *EP402R* gene encoding the CD2v protein [27] was amplified and sequenced using two sets of primers to generate two overlapping fragments. The primers used for the diagnosis and genotyping are presented in the Table 1.

**Table 1:** Primer used for diagnosis and molecular characterization of ASFV

Primer name	Primer sequences 5′ to 3′	Amplicon size (bp)	Ref.	Target gene or region
PPA1 / PPA2	F- AGTTATGGGAAACCCGACCC	257	[33]	B646L
	R- CCCTGAATCGGAGCATCCT			
P72_D / P72_U	F- GGCACAAGTTCGGACATGT	478	[15]	B646L
	R- GTACTGTAACGCAGCACAG			
PPA722 / PPA89	F- CGAAGTGCATGTAATAAACGTC	676	[11]	E183L
	R- TGTAATTTCATTGCGCCACAAC			
CVR–FL1 / CVR–FL2	F- TCGGCCTGAAGCTCATTAG	358	[24]	B602L
	R- CAGGAAACTAATGATGTTCC			
ECO1A / ECO1B	F- CCATTTATCCCCCGCTTTGG	356	[34]	*I73R* and *I329L*
	R- TCGTCATCCTGAGACAGCAG			
ga3611F / ga4220R	F- TATAATATAACAAATAATTGTAG	500	[27]	EP402R
	R- AGGGACGCATGTAGTAAATAG			
ga4124F / ga4698R	F- CTGAATCTAATGAAGAAGA	500	[27]	EP402R
	R- AAGTCTTTGTAGGTTTTTCGTTCA			

*Key*: *F* forward primer, *R* reverse primer, *Ref.* reference

PCR amplicons were confirmed using 2% agarose gel electrophoresis in the presence of molecular weight markers. For sequencing, PCR products were purified using Quick PCR purification Kit (QIAGEN, USA) following the manufacturer’s instructions and sent to Macrogen Europe BV (Amsterdam, The Netherlands) for Sanger sequencing. Open reading frames present within the sequences generated from the amplified CVR DNA fragments were translated into amino acid sequences using EMBOSS-Transeq software [19].

Both strands of purified amplicons were sequenced using the 7 primer sets for genotyping described above. To verify similarity with known sequences, the amplicon sequences obtained were submitted to BLAST (Basic Local Alignment Search Tool) [35] against non-redundant GenBank database. Multiple sequence alignments of sequences were generated using CLUSTAL W [36], whereas for each locus, the unrooted Maximum Likelihood method (ML) phylogenetic tree with 1000 bootstrap replications was estimated by MEGA 7 program and Kimura 2-parameter model [37] was selected based on the Best-Fit Substitution Model (ML) with the lowest Bayesian Information Criterion (BIC) score. ASFV sequence data of strains and isolates available in the GenBank were included as references. Sequences from this study have been submitted to GenBank with accession numbers MN689307 to MN689322 (for p72) and MN704903 to MN704917 (for p54).

## Results

### Detection of ASFV infection in the study locations

A total of 391 blood samples collected from symptomatic pigs were screened for the presence of the ASF viral DNA using conventional PCR with the diagnostic primers PPA1/PPA2. A total of 26 blood samples showed clear amplicons of the expected size (257 bp) and data were distributed as shown in Table 2, with the highest number of positive samples found in the Uvira territory 9/68 (13.2%), while the lowest was in Mwenga 2/65 (3.07%).

**Table 2:** ASFV PPA-PCR positive symptomatic pigs and proportion of P72 genotypes in selected territories of the South Kivu province, Eastern DRC

Territories	No. samples tested	PPA-PCR positive n(%)	P72 genotype X strains^**a**^	PCR assays		
				P72	P54	CVR
Fizi	61	4 (6.6)	Fizi-121	+	–	+
			Fizi-122	+	+	+
Kabare	66	3 (4.5)	Kabare-146	+	–	–
			Kabare-30	+	+	+
			Kabare-385	+	+	+
Kalehe	64	2 (3.1)	Kalehe-11	+	+	+
			Kalehe-49	+	+	+
Mwenga	65	2 (3)	Mwenga-119	+	+	+
			Mwenga-336	+	+	+
Uvira	68	9 (13.2)	Uvira-10	+	+	+
			Uvira-12	+	+	+
			Uvira-13	+	–	–
			Uvira-154	+	+	–
			Uvira-48	+	+	+
			Uvira-50	+	+	+
			Uvira-53	+	+	+
Walungu	67	6 (8.9)	Walungu-244	+	+	+
			Walungu-318	+	+	–
			Walungu-326	+	–	+
Total	391	26 (6.6)		19	15	15
No. strains positive for ASFV using the 3 PCR assays				13		

^a^PCR assays were performed on the 26 samples positive for PPA PCR

### Sequence analysis of ASFV based on the *B646L* (p72), *E183L* (p54) and *B602L* (CVR) genes

**Fig. 2:**
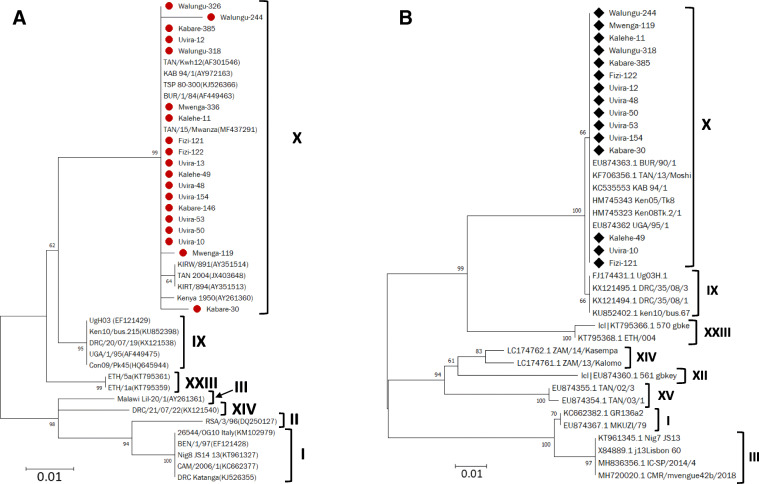
Phylogenetic relationships of p72 and p54 genotypes. The evolutionary history was inferred by the maximum likelihood method based on the Kimura 2-parameter model [33]. Phylogeny was inferred following 1000 bootstrap replications, and the node values show percentage bootstrap support. Scale bar indicates nucleotide substitutions per site. Scale bar indicates nucleotide substitutions per site. **a** p72 genotypes. The analysis included 19 *B646L* (p72) sequences from this study (plain circle ) and sequences from the GenBank database. The GenBank accession numbers for the different *B646L* (p72) genes are indicated in parenthesis. **b** p54 genotypes. The analysis involved 15 *E183L* (p54) gene sequences of African swine fever viruses from this study (black diamond ◆) and sequences from the GenBank database. The sequences for the different *B646L* (p72) and *E183L* (p54) genes are starting by GenBank accession numbers

Of the 26 ASFV positive samples using PPA diagnostic primers, we successfully amplified and sequenced 19 (73.07%) samples for p72 and 15 (57.69%) samples for p54 (Table 2). The p72 amplicon sequences shared 99–100% identity due to some few synonymous mutations while the p54 sequences were 100% identical (data not shown). Thus, CLUSTAL protein sequence alignment showed 100% identity between all the p72 and p54 sequences in the study samples. Sequences of p72 and p54 amplicon were compared with 25 other p72 and p54 ASFV sequences retrieved from the GenBank database and the phylogenetic analysis revealed that the South Kivu ASF virus strains analyzed clustered with p72 genotype X including strains reported in previous studies in Burundi (AF449463), Kenya (AY261360) and Tanzania (JX403648, AF301546, MF437291) (Fig. 2a and b). This is the first report of genotype X in the DRC.

In addition, the predicted amino acid sequences of the CVR nucleotide sequences were generated from 15 samples (2 Fizi, 2 Kabare, 2 Kalehe, 2 Mwenga, 5 Uvira, 2 Walungu) and specific features based on the previously reported ASFV tetrameric amino acid repeats within the CVR [38, 39] were obtained. Analysis of the CVR signature of the *B602L* gene showed two different signatures when compared with sequences of strains of the same genotype from Burundi, Tanzania and Uganda. All ASFV strains contained a CVR with 3 tetrameric amino acids, namely CAST (A), CADT (B) and NVDT (N). However, in strains from the Uvira territory the CVR sequence was repeated 10 times with the profile AAAABNAABA. In contrast, in strains from the five other territories, the CVR sequences contained only 8 repeats with the signature AABNAABA (Table 3). Both CVR signatures were different from the strains TAN13/Moshi, BUR 84/1, BUR84/2 and Ug95/3 [38–40].

**Table 3:** The amino acid sequence of the tetrameric repeats that constitute the central variable region (CVR) of the *B602L* gene characterized in this study

Strains	GenBank Acc. No.	CVR amino acid sequence	No. of TRS	P72 genotype	Reference
Fizi-121	MN689316	AABNAABA	8	X	This study
Fizi-122	MN689315	AABNAABA	8	X	This study
Kabare-30	MN689320	AABNAABA	8	X	This study
Kabare-385	MN689314	AABNAABA	8	X	This study
Kalehe-11	MN689308	AABNAABA	8	X	This study
Kalehe-49	MN689307	AABNAABA	8	X	This study
Mwenga-119	MN689310	AABNAABA	8	X	This study
Mwenga-336	MN689321	AABNAABA	8	X	This study
Uvira-12	MN689319	AABNAABA	8	X	This study
Walungu-244	MN689309	AABNAABA	8	X	This study
Walungu-326	MN689317	AABNAABA	8	X	This study
Uvira-10	MN689318	AAAABNAABA	10	X	This study
Uvira-48	MN689312	AAAABNAABA	10	X	This study
Uvira-50	MN689311	AAAABNAABA	10	X	This study
Uvira-53	MN689313	AAAABNAABA	10	X	This study
Uganda 95/3^a^	AM259420	AABNBABA	8	X	[40]
Tanzania/13Moshi^a^	KF706364	BNBBNBNNA	9	X	[38]
Burundi 84/1^a^	AM259422	AAAAAAABA	9	X	[39]
Burundi 84/2^a^	AM259423	AAAAAAABA	9	X	[39]

Key: A (CAST); B (CADT), and N (NVDT)

*CVR* central variable region, *TRS* tetrameric repeat sequence

^a^indicates strains retrieved from the GenBank and used as reference for comparison

### Sequence analysis of the intergenic region between *I73R* and *I329L* genes and the *EP402R* gene

**Fig. 3:**
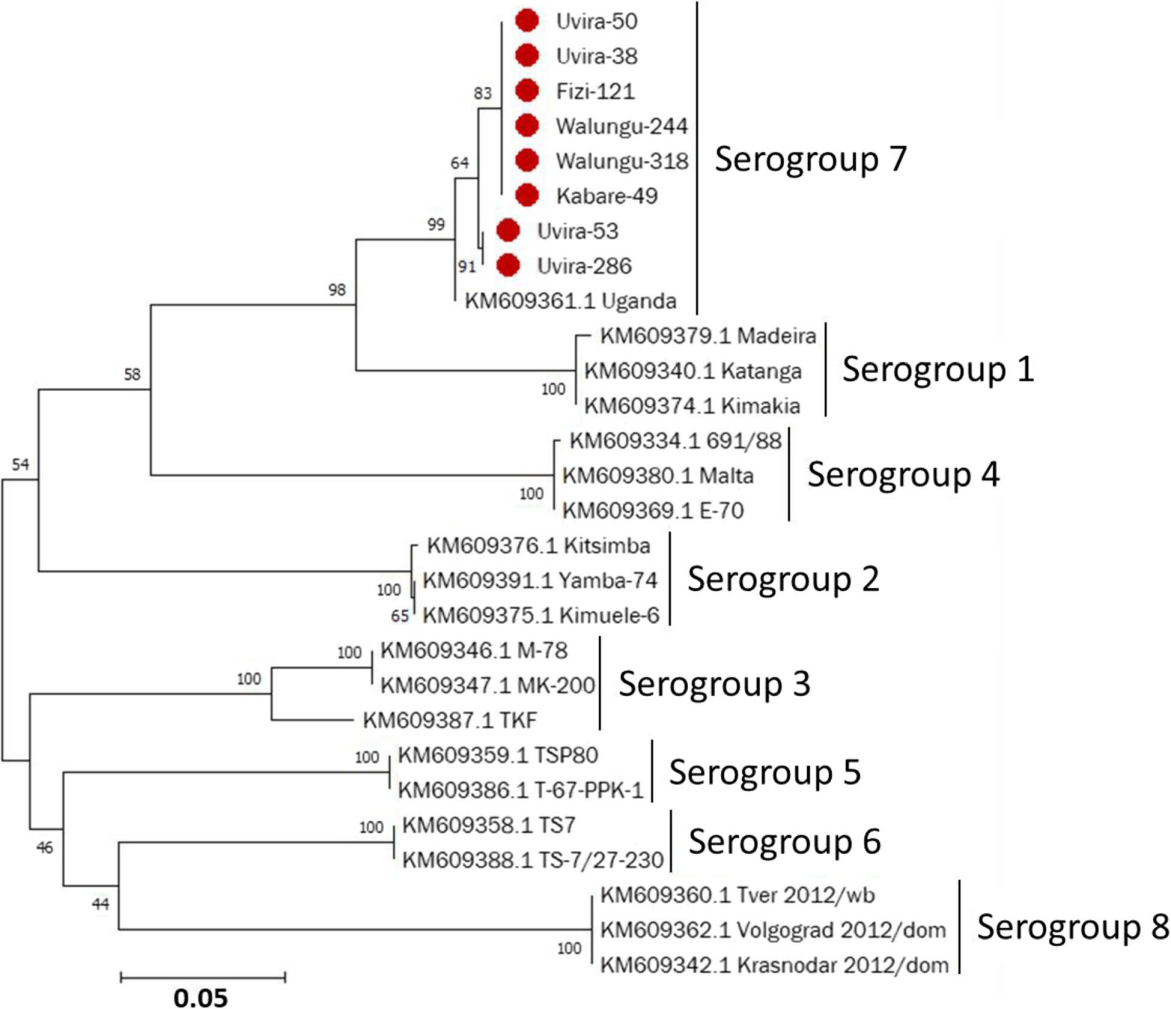
Maximum likelihood trees of ASFV CD2v protein sequences using the Kimura 2-parameter model [33]. Phylogeny was inferred following 1000 bootstrap replications, and bootstrap values greater than 50 are indicated at appropriate nodes. Scale bar indicates amino acid substitutions per site. The 8 sequences from this study are shown in plain circle (). Serogroup status of typed viral taxa are indicated

Amplification of the *EP402R* gene (encoding CD2V protein) was performed, and PCR amplicons of 8 strains from 4 territories were successfully sequenced. Comparative analysis of the 8 sequences obtained was carried out together with 20 other ASFV sequences retrieved from the GenBank database and previously characterized as serogroups. In this study, the phylogenetic analysis showed that the South Kivu strains belonged to serogroup 7 and were grouped with the Uganda strain (KM609361), the only available serogroup 7 in the GenBank. This research suggests that the strains from this study may have a similar hemadsorption inhibition (HAI) characteristics as the only known strain serogroup 7 (Fig. 3).

**Fig. 4:**
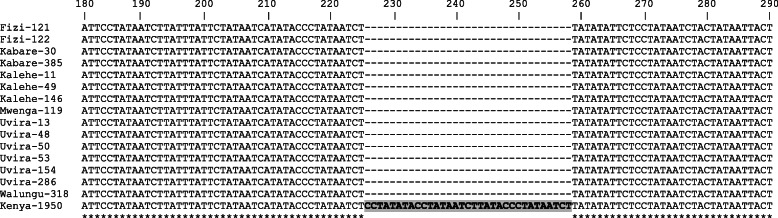
Partial nucleotide sequence alignments of the intergenic region between *I73R* and *I329L* genes. Sequences of African swine fever virus (ASFV) strains from the South Kivu province, eastern DRC, showing tetrameric repeats of representative genotypes**,** including a reference sequence of a virus isolated in 1950 in Kenya (Kenya 1950; GenBank accession no. AY261360.1). The indel that results from the insertion of the nucleotide sequence CCTATATACCTATAATCTTATACCCTATAATCT in the ASFV from Kenya is boxed

The analysis of whole-genome sequences of ASFV has facilitated identification of several regions containing tandem repeat sequences, important for discriminating between closely related ASFV strains and for predicting the origin of the virus [26]. In this study, the intergenic region between the *I73R* and *I329L* genes was analyzed for 15 strains from the 6 territories studied. The South Kivu ASFV strains were compared to the Kenyan 1950 strain (AY261360), which was identified from a domestic pig. The sequence alignment showed an indel of 33 bp (5′-CCTATATACCTATAATCTTATACCCTATAATCT-3′) between nucleotide position 226 to 258 (Fig. 4).

## Discussion

African swine fever constitutes the major obstacle to the development of the pig industry in the DRC, with sporadic outbreaks occurring across various areas throughout the year [24]. Despite recurrent occurrence of suspected ASF outbreaks in South Kivu province, information on the virus characterization remains scarce. To determine the prevalence of ASF and genotypes of ASFV in circulation in the South Kivu province, a study was carried out in the South Kivu province from January to August 2016, a period with no report of ASF outbreaks or cases in the sampled area [31]. We conducted a cross-sectional study in 5 of the 8 districts of the province and 267 pig blood from 250 smallholder pig farms were screened for presence of the ASFV antibody and viral genome using indirect Enzyme Linked Immunosorbent Assay (ELISA) and polymerase chain reaction (PCR), respectively, on asymptomatic domestic pigs. We found that 37% of pigs contained ASFV antibodies whereas virus DNA was present in 22.8% of pigs. Sequence analysis revealed that all the ASFV detected from asymptomatic pigs belonged to the genotype IX. Continuous characterization of ASFV strains is key in endemic regions to better understand disease outbreak patterns and map the different strains according to their geographical regions, in which they circulate [41].

The present study was targeting domestic pigs showing ASF clinical signs with the aim to characterize ASFV in symptomatic animals in the South Kivu province. We confirmed the presence of ASFV in domestic pigs with clinical signs of ASF in the six studied territories of South Kivu province: Kabare, Kalehe, Fizi, Mwenga, Uvira and Walungu. Although this study was not designed to determine the prevalence of ASFV, low rate of infection was observed in Mwenga and Kalehe (3 and 3.1%, respectively), whereas the highest infection rates were registered in Walunga and Uvira (9 and 13.2%, respectively). In our previous study which included asymptomatic pigs [31], Walungu had the highest prevalence of ASFV (33.7%) while the lowest ASFV prevalence was found in Kalehe (15.8%). The overall low infection rate may be attributed to the sensitivity of the assay used. Indeed, the conventional PCR method used in this study is less sensitive than other molecular methods such as nested-PCR [42] and real-time PCR [43] and may fail to detect potentially positive samples containing very low amount of viral genetic material. In addition to the low sensitivity of the conventional PCR used, other conditions may have contributed to the observed low prevalence including: (i) most pigs sampled may not have been infected or may have low virus load not detectable by the PCR technique used; and (ii) suspected pigs may have been affected by other diseases and conditions with similar symptoms to ASF such as porcine reproductive an respiratory syndrome, porcine dermatitis and nephropathy syndrome, salmonellosis. Our results confirmed ASF viral infections in pigs with clinical signs of ASF in the South Kivu province.

From the 26 PPA-PCR positive samples, 19, 15 and 15 samples were successfully amplified and sequenced for *B646L* (p72), *E183L* (p54) and *B602L* (CVR) genes, respectively. The combination of these three viral regions is to ensure a high-level resolution for ASFV discrimination. The p54 genotyping study corroborated the p72 analysis. Both P72 and P54 phylogenetic analyses clustered ASFV strains in circulation in symptomatic domestic pigs during the December 2018 – January 2019 outbreaks in the South Kivu province with ASFV genotype X, which is the major genotype associated with ASF outbreaks throughout Burundi, in some parts of Tanzania, Kenya and Uganda [38, 39]. Genotype X has been reported to be a sylvatic cycle-associated genotype that include ASFV identified from domestic pigs, warthogs as well as ticks in these three countries [15, 27, 44]. Furthermore, alignment of the 433 bp long sequence from the variable 3′-end of the *B646L* (p72) gene in the South Kivu viruses showed 100% identity with ASFV strains from Burundi 1984 (Data not shown). It is a possibility that viruses in this study may originate from, or could have expanded to Burundi. This scenario seems plausible as the South Kivu province is bordering Burundi through the river Rizizi and Lake Tanganyika, and uncontrolled cross-border movements of pigs and pork products are observed in the region and may constitute a major route of transmission of ASF in this endemic area [45]. Our current result contrasts with our previous finding of circulating ASFV strains of genotype IX in asymptomatic domestic pigs in the studied area during a period with no ASF outbreaks or cases [31]. It is unlikely that data from both studies suggest that ASFV of genotype IX may not cause disease in pigs whereas genotype X virus may cause ASF disease in domestic pigs in the South Kivu province of DRC, but it may have to do with the actual strains in circulation. Indeed, a screening of carcasses from outbreaks collected between 2005 and 2012 reveals co-circulation of strains of genotypes I, IX and XIV within DR Congo [40]. Although the report did not have any cases in the South Kivu province, it identified ASFV of genotype I in the neighboring province of Maniema. Nevertheless, further investigation in relation to both the host and virus genetics will be important to understand our findings. We are currently working on the lab-isolation of viruses of genotype IX and X in circulation in asymptomatic pigs and symptomatic pigs, respectively, for complete genome sequencing and comparative genomic analysis. Data obtained will improve our understanding of this contrasting finding in pigs within the South Kivu province. To the best of our knowledge, this is the first report of ASF virus genotype X in the DR Congo.

As all the strains were p72/p54 genotype X, we further characterized them at a higher resolution using the intra-genotypic central variable region (CVR) of the *BL602L* gene. Based on the tetrameric repeat sequences (TRS), our analysis identified two different CVR variants, clustering the strains into two subgroups. Subgroup 1 was composed only of strains from Uvira characterized by 10 TRS whereas all other strains formed the subgroup 2 and had only 8 TRS. The profile of the subgroup 2 (AABNAABA) was almost identical to the CVR amino acid sequence of Uganda 95/3 (AABNBABA), having the B code (CADT) in place of A at the 5th repeat [39]. The number of TRS repeat observed is relatively small compared to reports from some studies in the same geographic region describing the TRS motif repeated over 20 to 50 times [38, 46]. However, Mulumba–Mfumu et al. also observed this sequence repeated only 5 or 6 times in some DR Congo strains [24]. The two CVR variants found in our study were different from the previously reported variants in DR Congo ASFV strains [40] and to any other known viruses causing outbreaks or ASF cases, thus suggesting that the ASFV genotype 10 in circulation in the South Kivu province of DR Congo identified in this study may be unique [45, 47].

Within the vaccine field, it has been suggested that protective immunity is serotype-specific, as defined by ASFV hemadsorption inhibition (HAI) serological assay, with viruses within a serogroup cross-protecting against one another [17, 18]. The HAI assay can be used to type ASFV of a given genotype into distinct and individual serogroups, based on the ASFV proteins CD2v (*EP402R*) and/or C-type lectin (*EP153R*). Thus, HAI-based serogroup classification has been suggested as a better correlate for in vivo cross-protection among strains compared to the p72 genotyping [17]. In our study, we obtained CD2v sequences of 8 strains from 4 territories and comparative sequence analysis revealed that they were all identical. Moreover, phylogenetic analysis showed that the Uganda strain (GenBank Acc. No. KM609361.1), which represents the only member of the serogroup 7, was closest related to the South Kivu viruses, suggesting that the ASFV strains, identified in this study, may belong to serogroup 7. The high bootstrap value of 99% grouping the South Kivu strains with the Uganda serogroup 7 and the fact that strains from this study showed 99.2% amino acid identity with the Uganda serotype 7 strain strongly support the genetic relatedness between these two groups. It is worth noting that strains of serogroups 1 and 2 have been reported in DRC [18]. Overall, our data showed that these South Kivu ASFV strains are serologically different from other strains reported so far.

Analysis of the intergenic region between the *I73R* and *I329L* genes has previously been used for distinguishing between closely related ASFVs [26]. Characterization of this intergenic region genes did not identify any genetic diversity among the South Kivu strains. However, all the 15 strains analyzed had high sequence identity with the Kenyan strain 1950 (GenBank Acc. No. AY261360) but lacked an insertion of 33 bp. Indels have also been reported in a similar analysis [26]. Altogether, our study provided evidence of circulating ASFV genotype X which were antigenically related to serogroup 7 in domestic pigs with clinical signs of ASF in eastern DRC. The genotyping approach was also supported with the HAI serotyping for improved diversity analysis and finer discrimination of ASFV strains. This represents the first report of ASFV genotype X in DRC.

## Conclusion

In this study, ASFV isolated from symptomatic domestic pigs in the South Kivu province of the Democratic Republic of Congo were characterized for the genetic diversity. All the ASFV strains analyzed in this study belonged to the p72 genotype X and the CD2v serotype 7. This is the first report of circulating ASFV genotype X in DRC. The genetic similarity of these strains suggests that they may originate from a common source. However, CVR tetrameric sequence repeat analysis clustered the strains into a subgroup with 10 TSR (Uvira strains) and a subgroup with 8 TRS (strains from other territories), thus underlining genetic variation among these ASFV. Therefore, a better understanding of ASFV evolution and spread throughout the South Kivu province will need further in-depth comparative sequence analyses including whole genome sequencing of ASFV strains circulating in the area.

## Data Availability

The datasets used and/or analyzed during the current study are available from the corresponding author on reasonable request.

## Abbreviations

ASF: African swine fever
ASFV: African swine fever virus
CVR: Central variable region
DRC: Democratic Republic of Congo
HAI: Hemadsorption inhibition
PAUISTI: Pan African University Institute of Science Technology and Innovation
PCR: Polymerase chain reaction
TRS: Tetrameric amino acid repeat sequence
UEA: Université Evangélique en Afrique
